# Gestational Weight Gain per Pre-Pregnancy Body Mass Index and Birth Weight in Twin Pregnancies: A Cohort Study in Wuhan, China

**DOI:** 10.1038/s41598-018-29774-z

**Published:** 2018-08-21

**Authors:** Yawen Chen, Yan Liu, Yiming Zhang, Ronghua Hu, Zhengmin Qian, Hong Xian, Michael G. Vaughn, Mingzhu Liu, Shiyi Cao, Yong Gan, Bin Zhang

**Affiliations:** 1 Wuhan Children’s Hospital (Wuhan Maternal and Child Healthcare Hospital), Tongji Medical College, Huazhong University of Science and Technology, No. 100 Hongkong Road, Wuhan, 430014 Hubei People’s Republic of China; 2 Department of Epidemiology and Biostatistics, College for Public Health and Social Justice, Saint Louis University, 3545 Lafayette Avenue, Saint Louis, MO 63104 United States of America; 3 School of Social Work, College for Public Health and Social Justice, Saint Louis University, Tegeler Hall, 3550 Lindell Boulevard, Saint Louis, MO 63103 United States of America; 4 Department of Social Medicine and Health Management, School of Public Health, Tongji Medical College, Huazhong University of Science and Technology, NO. 13 Hangkong Road, Wuhan, 430030 Hubei People’s Republic of China

## Abstract

To assess the relationship between gestational weight gain (GWG) of twin-pregnancy women and twin birth weights, as well as to evaluate whether pre-pregnancy body mass index (BMI) influences this relationship. A cohort study was conducted in Wuhan, China, between 1/01/2011 and 8/31/2017. Women with twin pregnancies who delivered live and non-malformed twins were included (6,925 women and 13,850 infants), based on the Wuhan Maternal and Child Health Management Information System. Logistic regression models were employed to examine the association between GWG and paired small for gestational age (SGA, defined as birth weight <10th percentile for gestational age and sex)/SGA and linear regression models were utilized to explore the relationship between GWG and sum of birth weights. The associations of GWG based on both the IOM and Chinese recommendations and SGA/SGA pairs were obtained, as well as the stratified analyses by pre-pregnancy BMI. Additionally, the sum birth weight of one twin pair increased by 15.88 g when the GWG increased by 1 kg. GWG below the IOM and Chinese recommendations was associated with an increased risk of SGA/SGA pairs in all pre-pregnancy BMI categories. However, in underweight, overweight, and obese women, the association between GWG above the IOM and Chinese recommendations and SGA/SGA pairs changed with adjustment.

## Introduction

Gestational weight gain (GWG) is an important indicator for monitoring and evaluating the nutritional status of pregnant women^1^. Women with excess GWG are more likely to retain high postpartum weight, which can cause long-term maternal obesity and subsequent pre-pregnancy obesity^2,3^. Simultaneously, women with twin pregnancies have greater GWG than women with singleton pregnancies and are more likely to experience cesarean deliveries, diabetes, and pre-eclampsia^4,5^. GWG not only affects mothers’ health, but also influences the growth and development of fetuses. Excessive GWG increases the risk of macrosomia^1^ and childhood obesity^6^. While inadequate GWG increases the risk for low birth weight (LBW), preterm birth, and small for gestational age (SGA) among singletons^1,7,8^, these outcomes are especially risky in twin pregnancies^9,10^.

Birth weight largely reflects the fetal growth and development. Not only does SGA increase the risk of neonatal death^11,12^, but also affects an infant’s development and subsequent disease occurrence^13,14^. One previous study reported that 47.2% of women with twin pregnancies delivered LBW infants^15^. Currently, twin pregnancies account for 2%–4% of all birth worldwide^16,17^ and that frequency is increasing^18^. Studies concerning GWG in twin pregnancies and birth weight have indicated that the likelihood of twins weighing >2500 g or >1500 g or appropriate for gestational age (AGA) were significantly higher for women gaining weight at or above guidelines^19–21^.

The greater proportion of preterm births occurring among twin pregnancies complicates the issue of attaining appropriate GWG. As early as 1990, the Institute of Medicine (IOM) recommended guidelines for GWG in full-term twin pregnancies, regardless of pre-pregnancy body mass index (BMI). With the growing knowledge that pre-pregnancy BMI affects optimal GWG in women with twin pregnancies^15^, the IOM revised its recommendations for GWG in twin-pregnancy women, as follows: 17–25 kg for normal weight women (BMI: 18.5–24.9 kg/m^2^), 14–23 kg for overweight women (BMI: 25.0–29.9 kg/m^2^), and 11–19 kg for obese women (BMI: ≥ 30 kg/m^2^)^6^. Although these recommendations were proposed on the assumption of a term delivery (37–42 gestational weeks) and described as “provisional”, they were still recommended in a systematic review of nutrition for twin pregnancies^22^.

Using the IOM recommendations as reference, many studies have investigated the association of GWG in twin-pregnancy women and pregnancy outcomes^15,19,20^. However, almost all of those studies were conducted in industrialized countries. A small number of studies have assessed this issue in non-industrialized countries, including China. Additionally, the Chinese adult BMI category standards for normal, overweight, and obese were lower than those found in the WHO^23^, and the IOM did not provide guidelines for underweight women because of insufficient information. In order to improve representativeness and provide more data, there is a critical need to expand sample size to evaluate the relationship of GWG in twin pregnancies and relevant outcomes.

Therefore, this cohort study was conducted and based on both the Chinese adult BMI categories and the IOM GWG recommendations in twin pregnancies to elucidate the association of GWG and birth weights. This association was then stratified by per pre-pregnancy BMI among a population of women with twin-pregnancy in Wuhan, China.

## Results

**Figure 1:**
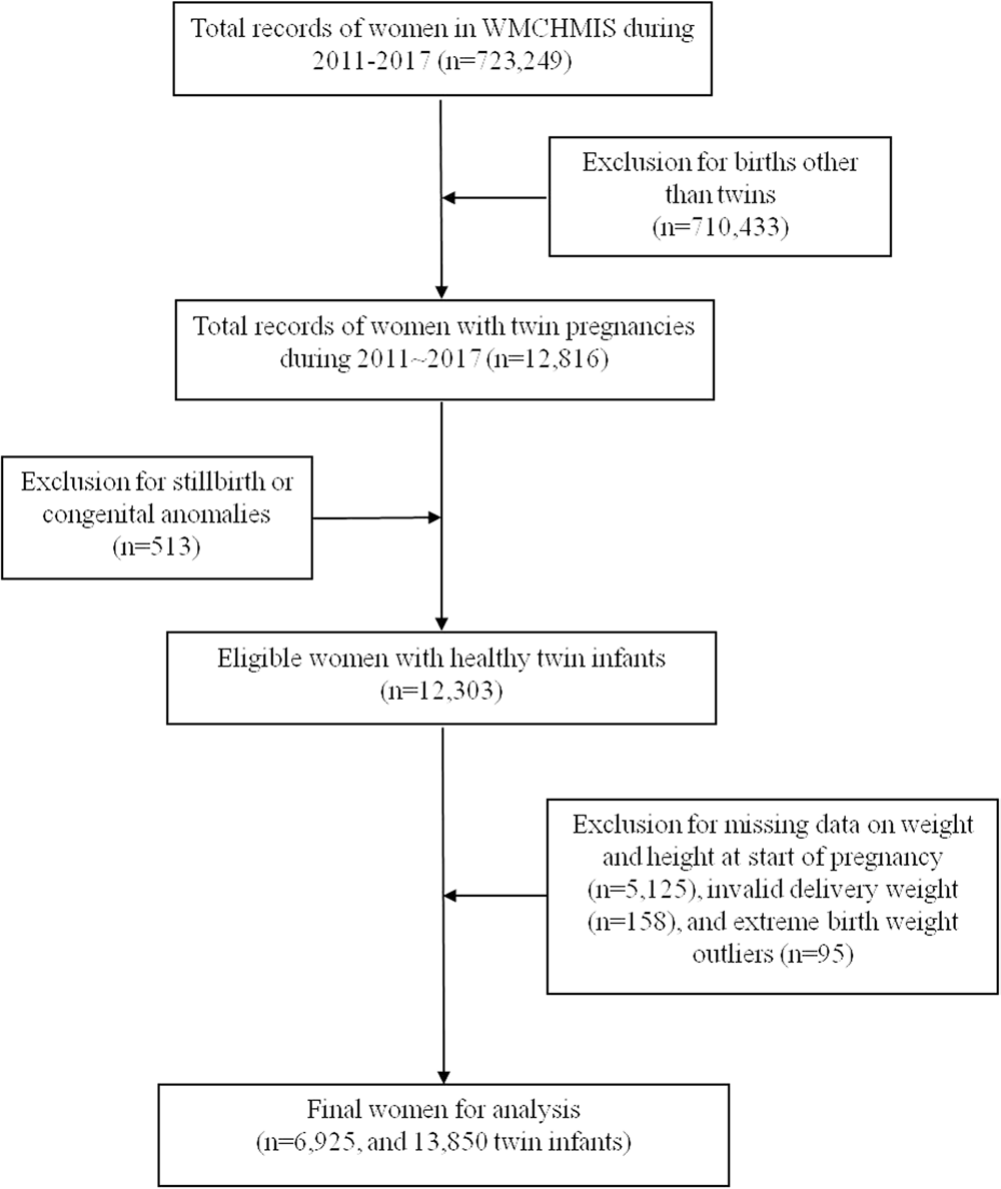
Flow chart of participant selection.

Data for 6,925 women, with twin pregnancies and meeting the study criteria, were analyzed (Fig. 1). According to pre-pregnancy BMI, 854 (12.33%) were classified as underweight, 5,225 (75.45%) were normal weight, 715 (10.32%) were overweight, and 131 (1.89%) were obese (Table 1). The differences between the IOM and Chinese recommended GWG by BMI groups were shown in Table 2. Table 3 presented participants characteristics. Of the 6,925 twin-pregnancy women, 39.26% were 25–29 years old and 53.90% were educated more than 12 years. Of included women, 67.06% gained weight at or above the IOM recommendations and 76.47% gained weight at or above the Chinese recommendations. 3,315 twin-pregnancy women delivered full-term twin infants and nearly two-thirds of the participants had the same sex twins. Birth weights were examined among twins, based on the IOM and Chinese recommended GWG, and presented in Table 4. 148 women (2.14%) delivered SGA/SGA paired. The mean birth weight of 6,925 twin pairs was 4910.95 g. About half of the women (55.58%) had normal weight twins and most of the women (85.66%) delivered concordant twin pairs. Twin-pregnancy women whose GWG met or exceeded the IOM and Chinese recommendations were more likely to have heavier and normal weight twins.

**Table 1:** Total GWG in Chinese women with twin pregnancies by different pre-pregnancy BMI.

Pre-pregnancy BMI	China BMI Standard (kg/m^2^)	No. of Subjects (%)	Total GWG (kg), mean ± SD	25th Percentile (kg)	75th Percentile (kg)	10th and 90th Percentiles (kg)
Underweight	<18.5	854 (12.33)	22.82 ± 7.44	18	26	15–31
Normal	18.5–23.9	5225 (75.45)	20.44 ± 8.15	15	25	11–31
Overweight	24–27.9	715 (10.32)	16.58 ± 7.05	12	21	7–25
Obese	≥28	131 (1.89)	14.79 ± 6.90	9	20	5–24

Abbreviations: GWG, gestational weight gain; BMI, body mass index; SD, standard deviation.

**Table 2:** The differences between the IOM and Chinese recommended GWG by pre-pregnancy BMI.

Pre-pregnancy BMI	China BMI Standard (kg/m2)	Chinese-recommended GWG (kg)	IOM BMI Standard (kg/m^2^)	IOM-Recommended GWG (kg)
Underweight	<18.5	18–26	<18.5	—
Normal	18.5–23.9	15–25	18.5–24.9	17–25
Overweight	24–27.9	12–21	25–29.9	14–23
Obese	≥28	9–20	≥30	11–19

Abbreviations: IOM, the Institute of Medicine.

**Table 3:** Characteristics of women and twin infants.

	N (%)
**Age at delivery**	
< = 19	38 (0.55)
20–24	812 (11.73)
25–29	2719 (39.26)
30–34	2479 (35.80)
≥35	877 (12.66)
**Education level**	
Less than 9 years	1525 (22.08)
9–12 years	1659 (24.02)
More than 12 years	3723 (53.90)
**Parity**	
Nullipara	5259 (75.94)
Multipara	1666 (24.06)
**Gravidity**	
<3	5465 (78.92)
≥3	1460 (21.08)
**Pre-pregnancy BMI (kg/m** ^**2**^ **)**	
Underweight(<18.5)	854 (12.33)
Normal (18.5–23.9)	5225 (75.45)
Overweight (24–27.9)	715 (10.32)
Obese (≥28)	131 (1.89)
**Total GWG**	Chinese-recommended/IOM- recommended
Low	1629 (23.52) 2000 (32.94)
Normal	3694 (53.34) 2714 (44.70)
High	1602 (23.13) 1357 (22.35)
**Birth type**	
Caesarean section	6507 (93.96)
Vaginal delivery	418 (6.04)
**Offspring sex**	
Male/male pairs	2427 (35.05)
Female/female pairs	2235 (32.27)
Male/female pairs	2263 (32.68)
**Apgar score 1 minute**	
≥7	13313 (96.12)
<7	537 (3.88)
**Apgar score 5 minute**	
≥7	13712 (99.00)
<7	138 (1.00)
**Gestational weeks**	
<28	7 (0.10)
28–32	373 (5.39)
33–36	3230 (46.64)
≥37	3315 (47.87)

**Table 4:** Birth weight by the IOM and Chinese recommended GWG among twins.

	Mean (±SD)/N (%)	Chinese recommended			*P*	IOM recommended			*P*
		Low GWG	Normal GWG	Excess GWG		Low GWG	Normal GWG	Excess GWG	
SGA/SGA pairs	148 (2.14)	65 (43.92)	58 (39.19)	25 (16.89)	<0.001	58 (53.21)	34 (31.19)	17 (15.60)	<0.001
SGA/AGA pairs	1054 (15.22)	285 (27.04)	587 (55.69)	182 (17.27)		324 (36.73)	410 (46.49)	148 (16.78)	
AGA/AGA pairs	5723 (82.64)	1279 (22.35)	3049 (53.25)	1395 (24.38)		1618 (31.85)	2270 (44.69)	1192 (23.46)	
Larger twin birth weight (g)	2599.61 (±411.17)	2451.62 (±450.13)	2607.03 (±387.30)	2731.04 (±373.40)	<0.001	2495.32 (±437.89)	2629.67 (±384.40)	2745.91 (±369.66)	<0.001
Smaller twin birth weight (g)	2311.79 (±411.21)	2183.87 (±433.19)	2314.52 (±397.38)	2435.57 (±379.77)	<0.001	2218.16 (±429.16)	2335.55 (±391.23)	2446.59 (±378.67)	<0.001
Sum birth weight of twins	4910.95 (±785.09)	4635.50 (±852.59)	4921.55 (±745.00)	5166.61 (±709.49)	<0.001	4713.48 (±833.22)	4965.22 (±736.35)	5192.50 (±703.98)	<0.001
Both twins > = 2500 g	2639 (55.58)	429 (16.26)	1409 (53.39)	801 (30.35)	<0.001	584 (24.70)	1092 (46.19)	688 (29.10)	<0.001
Both twins > = 1500 g and <2500 g	2005 (42.23)	637 (31.77)	1040 (51.87)	328 (16.36)		697 (41.39)	734 (43.59)	253 (15.02)	
Both twins >= 1000 g and <1500 g	104 (2.19)	54 (51.92)	45 (43.27)	5 (4.81)		57 (62.64)	30 (32.97)	4 (4.40)	
**Twin concordance**									
Concordant twin pairs	5932 (85.66)	1405 (23.69)	3144 (53.00)	1383 (23.31)	0.377	1720 (33.10)	2307 (44.39)	1170 (22.51)	0.348
Discordant twin pairs	993 (14.34)	224 (22.56)	550 (55.39)	219 (22.05)		280 (32.04)	407 (46.57)	187 (21.40)	

Abbreviations: SGA, small for gestational age; AGA, appropriate for gestational age.

ORs for SGA/SGA pairs by GWG and pre-pregnancy BMI were described in Table 5. Based on the IOM and Chinese guidelines, GWG below recommendations was associated with an increased risk for SGA/SGA pairs in twin-pregnancy women (IOM: OR = 2.39, 95% CI = 1.56–3.67, *P* < 0.0001; Chinese: OR = 2.67, 95% CI = 1.86–3.83, *P* < 0.0001). Although it was not statistically significant, a trend towards lower risk for SGA/SGA pairs in twin-pregnancy women with GWG above recommendations was observed (IOM: OR = 0.95, 95% CI = 0.53–1.71, *P* = 0.8699; Chinese: OR = 0.94, 95% CI = 0.59–1.51, *P* = 8049). The relationship persisted after adjusting for aforementioned confounders.

**Table 5:** ORs of SGA pairs in relation to GWG and pre-pregnancy BMI.

Pre-pregnancy BMI (kg/m^2^)		NO. of SGA pairs/NO. of AGA pairs	Crude OR (95% CI)	P value	Adjusted OR (95% CI)	P value
**Total GWG**						
Low	IOM	65/1279	2.39 (1.56, 3.67)	<0.0001	2.53 (1.64, 3.91)	<0.0001^*^
	Chinese	58/1618	2.67 (1.86, 3.83)	<0.0001	2.92 (2.02, 4.21)	<0.0001^*^
Normal	IOM	58/3049	Reference		Reference	
	Chinese	34/2270				
High	IOM	25/1395	0.95(0.53, 1.71)	0.8699	0.90 (0.50, 1.62)	0.7215^*^
	Chinese	17/1192	0.94 (0.59, 1.51)	0.8049	0.88 (0.55, 1.42)	0.6038^*^
**Underweight (**<**18.5 kg/m**^**2**^**)**						
Low GWG	IOM	—	—	—	—	—
	Chinese	16/118	3.45 (1.63, 7.23)	0.0012	4.96 (2.25, 10.93)	<0.0001^§^
Normal GWG	IOM	—	—	—	—	—
	Chinese	14/356	Reference			
High GWG	IOM	—	—	—	—	—
	Chinese	9/169	1.35 (0.58, 3.19)	0.4881	1.37 (0.57, 3.30	0.4839
**Normal (18.5~23.9 kg/m** ^**2**^ **)**						
Low GWG	IOM	50/1387	2.14 (1.36, 3.35)	0.0009	2.30 (1.46, 3.63)	0.0004^§^
	Chinese	42/937	2.63 (1.70, 4.08)	<0.0001	2.80 (1.79, 4.38)	<0.0001^§^
Normal GWG	IOM	32/1897	Reference		Reference	
	Chinese	40/2347				
High GWG	IOM	14/1054	0.79 (0.42, 1.48)	0.459	0.75 (0.40, 1.42)	0.3835^§^
	Chinese	14/1054	0.78 (0.42, 1.44)	0.4254	0.74 (0.40, 1.36)	0.3286^§^
**Overweight and obese (**> = **24 kg/m**^**2**^**)**						
Low GWG	IOM	2/231	6.46 (1.36, 30.68)	0.0190	5.59 (1.22, 28.25)	00276^§^
	Chinese	7/224	2.70 (0.79, 9.34)	0.1159	2.45 (0.70, 8.62)	0.1615^§^
Normal GWG	IOM	2/373	Reference		Reference	
	Chinese	4/346				
High GWG	IOM	3/138	4.06 (0.67, 24.52)	0.1274	3.43 (0.56, 21.02)	0.1830^§^
	Chinese	2/172	1.01 (0.18, 0.55)	0.9947	0.94 (0.17, 5.25)	0.9455^§^

Abbreviations: OR, odds ratio; CI: confidence interval; SGA, small for gestational age; AGA, appropriate for gestational age.

^*^Adjusted for maternal delivery age (continuous), education level, parity, gravidity, pre-pregnancy BMI, sex of twin infants, and gestational weeks.

^§^Adjusted for maternal delivery age (continuous), education level, parity, gravidity, sex of twin infants, and gestational weeks.

In the subsequent stratified analyses, pre-pregnancy BMI modified the associations between GWG and SGA/SGA pairs on the basis of the IOM and Chinese recommendations. Using the Chinese recommended GWG as a reference, low GWG was associated with a significantly increased risk for SGA/SGA pairs in the underweight group (OR = 4.96, 95% CI = 2.25–10.93, *P* < 0.0001), the normal pre-pregnancy BMI group (OR = 2.80, 95% CI = 1.79–4.38, *P* < 0.0001), but was not significant in the overweight and obese group (OR = 2.45, 95% CI = 0.70–8.62, *P* = 0.1615). The associations between high GWG and SGA/SGA pairs were negative in the normal pre-pregnancy BMI women (OR = 0.74, 95% CI = 0.40–1.36, *P* = 0.3286), and overweight and obese women (OR = 0.94, 95% CI = 0.17–5.25, *P* = 0.9455), but was positive in the underweight women (OR = 1.37, 95% CI = 0.57–3.30, *P* = 0.4839). Using the IOM recommended GWG as a reference, low GWG resulted in a positive relationship for SGA/SGA pairs in normal pre-pregnancy BMI women (OR = 2.30, 95% CI = 1.46–3.63, *P* = 0.0004) and overweight and obese women (OR = 5.59, 95% CI = 1.22–28.25, *P* = 0.0276). High GWG resulted in a non-significantly negative association for SGA/SGA pairs in the normal pre-pregnancy BMI group (OR = 0.75 95% CI = 0.40–1.42, *P* = 0.3835), but was positive in the overweight and obese group (OR = 3.43, 95% CI = 0.56–21.02, *P* = 0.1830).

The associations between GWG and SGA/AGA pairs were provided in Supplementary Table S1.

The results of linear regression analyses were shown in Table 6. After adjusting for gestational weeks, delivery age, pre-pregnancy BMI and parity, the sum birth weight of a twin pair increased by 15.88 g when the GWG increased by 1 kg.

**Table 6:** Results of linear regression analyses for the sum birth weight of a twin pair.

Values	Parameter estimate	Standard error	F value	P value
Intercept	−6593.26	143.44	2112.86	<0.0001
Gestational weeks (week)	275.86	3.46	6361.65	<0.0001
Gestational weight gain (kg)	15.88	0.85	348.28	<0.0001
Delivery age (year)	13.32	1.51	78.08	<0.0001
Pre-pregnancy BMI (kg/m^2^)	36.13	2.66	184.93	<0.0001
Parity	63.44	15.67	16.38	<0.0001

## Discussion

Given the dearth of evidence surrounding the association of GWG in women with twin pregnancies in large sample and in non-western industrialized nations, the present study sought to fill this gap. This study revealed that GWG below the IOM and Chinese recommendations was associated with an increased risk for SGA/SGA pairs.

Previous studies have reported that GWG in twin-pregnancy women was related to birth weights^24–28^. The results of this current study were similar to one previous study. Olha Lutsiv *et al*. studied 741 women with twin pregnancies and found that GWG irrespective of maternal pre-pregnancy BMI, below the IOM guidelines was a risk factor for SGA (OR = 1.44, 95% CI = 1.01–2.06), and GWG above the IOM guidelines tended to be a protective factor for SGA (OR = 0.92, 95% CI = 0.62–1.36)^21^. Since GWG is an important indicator of pregnant women’s nutritional status, it is of importance in the optimization of neonatal outcomes and reduction of SGA. As GWG and nutrition are influential factors for pregnancy outcomes, this information is important for both patients and clinicians.

We also conducted stratified analyses by pre-pregnancy BMI, which found that there was no difference between the associations of SGA/SGA pairs and GWG according to both the IOM and the Chinese recommendations in normal pre-pregnancy BMI women. However, in the overweight and obese women, weight gain greater than the Chinese recommended GWG decreased the risk for SGA/SGA pairs, but increased the risk for SGA/SGA pairs according to the IOM recommended GWG. A previous study was conducted in America and showed that in the overweight and obese groups, there was no difference between SGA incidence and GWG at or above the IOM recommendations^29^, which was consistent with another study^25^. The differences found among these studies may be attributed to the study population and BMI classification standard mentioned above.

Additionally, the results of this study revealed that the sum birth weight of a twin pair increased with an increase in GWG. One other study, Chu *et al*. reported that the adjusted percentage of normal birth weight infants generally increased with increasing GWG in each BMI group^15^.

Multiple cohorts of twin pregnancies have utilized the 2009 IOM revised guidelines to test their effectiveness. However, only data from women in industrialized countries were used by the IOM to assess GWG. Thus, the IOM guidelines were widely used in the United States and Europe. Additionally, the IOM did not provide guidelines for underweight women due to insufficient information. Therefore, this study, which was conducted in China and included data from underweight women, is useful. This study included 854 underweight women with twin pregnancies. It showed that, in this group, GWG both below and above the Chinese recommendations increased the risk for SGA/SGA pairs. Further research is warranted in underweight women with twin pregnancies to identify the optimal GWG and to improve pregnancy outcomes.

At present, there is not yet an official GWG recommendation in twin-pregnancy women in China. In this study, 53.21% of women who gained below the IOM recommendations delivered SGA/SGA pairs, and 43.92% of women who gained below the Chinese recommendations delivered SGA/SGA pairs. These results support the view that the IOM revised recommendations may be suitable for Chinese twin-pregnancy women, which was inconsistent with a previous study focused on Chinese women with singleton pregnancies^1^. The results of this study also demonstrated that twin-pregnancy women within GWG recommendations have larger neonates. Thus, these recommendations seem effective and deserve attention in clinical practice. Nutritional interventions for twin pregnancies could be built into future studies to evaluate their impact on birth weight. Moreover, these interventions could provide a simple way to reduce morbidity and mortality related to SGA.

This cohort study possessed multiple strengths. It was the first study to investigate the association between GWG and birth weights in Chinese twin-pregnancy women and to explore this association with stratification by pre-pregnancy BMI. The association of GWG and birth weights in underweight twin-pregnancy women was also analyzed, which is an understudied yet important topic. Additionally, several significant associations emerged, and the merit of these associations is clinically noteworthy. However, despite these study assets, there are three notable limitations. First, information on women with twin pregnancies who visited hospitals for prenatal examination and did not give information to obstetricians in community health centers was not obtained. Twin-pregnancy women who directly visit hospitals for prenatal examination may have higher socioeconomic status and gain more weight. Therefore, the extension of these results to all twin-pregnancy women should be performed with caution. Second, BMI values are more likely to be underestimated because individuals tend to underreport their weight and overreport their height^30,31^. Finally, we could not obtain the information of chorionicity and amnionicity of twins, which may affect the twin birth weight.

In conclusion, a GWG below the IOM and Chinese recommendations increased the risk of SGA/SGA pairs. This association occurred in all BMI categories. Due to these associations, nutritional assessment and counseling must be part of prenatal care. Additionally, in underweight, overweight, and obese women, the association between GWG above the IOM and Chinese recommendations and SGA/SGA pairs changed following adjustments. This indicates that the relationship between GWG and birth weights in twin-pregnancy women with an abnormal pre-pregnancy BMI needs to be further evaluated.

## Materials and Methods

### Ethics approval and informed consent

The methods were performed in accordance with the approved guidelines and regulations. The Ethics Committee of Wuhan Children’s Hospital (Wuhan Maternal and Child Healthcare Hospital) approved this study. Before participating in the study, each woman received a written informed consent.

### Study population

The cohort study was conducted in Wuhan, a large city of approximately 10 million people in central China. This study was based on the Wuhan Maternal and Child Health Management Information System (WMCHMIS), which was constructed to improve the monitoring of pregnancy outcomes by Wuhan Maternal and Child Healthcare Hospital. Additional information can be found in a previous study^32^. The data for the current study were obtained from this system between January 1, 2011 and August 31, 2017.

Women enrolled in this study included those who delivered live twin newborns with no congenital malformation after more than 26 gestational weeks during the aforementioned specified timeframe. Women who delivered stillbirth or births with congenital anomalies (n = 513) were excluded. Those with missing data on weight and height in the beginning of pregnancy (n = 5125) and invalid delivery weight (defined as delivery weight less than pre-pregnant weight) (n = 158) were also excluded. Additionally, extreme birth weight outliers (n = 95) (defined as values of the mean twins’ weight minus or plus three-time standard deviation (SD)) were eliminated. In total, 6,925 women with twin pregnancies and 13,850 infants were included in this study.

### Variables

Maternal demographic characteristics including delivery age, education level, gravidity, parity, pre-pregnancy weight and height were self-reported at the first visit to community health centers for antenatal examination. Gestational age was identified by the date of the last menstrual period, and confirmed by B-ultrasound. Delivery and neonatal information was recorded in the WMCHMIS by midwives, and audited by obstetricians and obstetric nurses. If illogical data which was defined by experts in gynecology and obstetrics (for example, the number of parity will be illogical if it is more than that of gravidity) was inputted to the WMCHMIS, a warning was activated.

Pre-pregnancy BMI was computed as: weight (in kilograms) divided by height squared (in square meters). It was then classified into four groups on the basis of the Chinese adult standards: underweight (<18.5 kg/m^2^), normal weight (18.5–23.9 kg/m^2^), overweight (24–27.9 kg/m^2^), and obese (≥28 kg/m^2^)^23^.

We subtracted pre-pregnancy weight from weight on delivery day to obtain total GWG. In accordance with the 2009 IOM Guidelines for GWG in twin pregnancies, recommended GWG for a normal pre-pregnancy BMI is 17–25 kg, overweight women, 14–23 kg, and obese women, 11–19 kg^6^. The Chinese recommended GWG was derived from the interquartile range (25^th^–75^th^ percentiles) of this sample, which was adopted by the IOM^6^.

Maternal delivery age was categorized into five groups: younger than 20, 20–24, 25–29, 30–34, and 35 years and older. Maternal education level was treated as a proxy for socioeconomic status and was categorized into three groups: less than 9 years, 9–12 years, and more than 12 years. Women were dichotomized by parity into nullipara and multipara. Women were also dichotomized by gravidity into <3 times and ≥3 times. Gestational weeks were divided into four groups: <28 weeks, 28–32 weeks, 33–36 weeks, and ≥37 weeks.

The main outcome variable was birth weight. Paired SGA was treated as dependent variable in this study. Newborns were classified as SGA if their birth weight was below the 10th percentile and as AGA if it was at or above the 10th percentile for gestational age and sex based on twin birth weight curves in Chinese twins^33,34^. The twin concordance of birth weights, defined as ≤20%, was determined by the following formula: [(Birth weight of larger one – Birth weight of smaller one)/Birth weight of larger one] × 100. The twin discordance of birth weights defined as >20% using the same formula mentioned above.

### Statistical analysis

Descriptive statistics were presented as means (±SDs) for continuous variables or frequencies (%) for categorical variables. Categorical and continuous variables were analyzed by chi-squared tests and variance, respectively. Several confounders were selected based on previous studies, including maternal delivery age (continuous), education level, pre-pregnancy BMI, parity, gravidity, GWG, sex of infants, and gestational weeks.

Logistic regression models were performed to assess the association of GWG and SGA/SGA pairs. A stratified analysis by pre-pregnancy BMI categories was also used and separated logistic regression models were established based on the Chinese and the IOM recommended GWG. Each logistic model included adjustments for confounders. Crude and adjusted odds ratios (ORs), as well as a 95% confidence interval (CI), were calculated. A linear regression analysis, with 0.1 inclusion and 0.15 exclusion criteria, was used to explore the relationship between GWG and the sum birth weight of a twin pair.

SAS V.9.2 (SAS Statistical Institute) was used to complete all statistical analyses.

### Data availability

The datasets analyzed during the current study are not publicly available due to patient privacy but are available from the corresponding author on reasonable request.

## Electronic supplementary material

Supplementary Table S1 ORs of SGA/AGA pairs in relation to GWG and pre-pregnancy BMI
