# Supplementary material for: Gestational Weight Gain per Pre-Pregnancy Body Mass Index and Birth Weight in Twin Pregnancies: A Cohort Study in Wuhan, China
Source: Sci Rep. 2018 Aug 21;8:12496. doi: 10.1038/s41598-018-29774-z (PMC6104075; doi:10.1038/s41598-018-29774-z)
Supplement: Supplementary file 1 — Supplementary Table S1 ORs of SGA/AGA pairs in relation to GWG and pre-pregnancy BMI [file 41598_2018_29774_MOESM1_ESM.pdf]

Supplementary legend

Supplementary Table S1 ORs of SGA/AGA pairs in relation to GWG and pre-pregnancy BMI

Gestational Weight Gain per Pre-Pregnancy Body Mass Index and Birth Weight in Twin Pregnancies: A Cohort Study in Wuhan, China

Yawen Chen[§], Yan Liu[§], Yiming Zhang, Ronghua Hu, Zhengmin Qian, Hong Xian, Michael G. Vaughn, Mingzhu Liu, Shiyi Cao, Yong Gan, Bin Zhang[*]

[§] These authors contributed equally to this work

[*] **Corresponding author**

Full name: Bin Zhang

Postal address: No.100 Hongkong Road, Wuhan 430014, Hubei, China,

E-mail: mchwhzb@163.com

Telephone and fax number: +86-027-82433149

Supplementary Table S1 ORs of SGA/AGA pairs in relation to GWG and pre-pregnancy BMI

Pre-pregnancy BMI (kg/m^2)		NO. of SGA/AGA pairs vs. NO. of AGA pairs	Crude OR (95% CI)	P value	Adjusted OR (95% CI)	P value
**Total GWG**						
Low	IOM	324/1618	1.11 (0.95, 1.30)	0.2035	1.16 (0.99, 1.36)	0.0719*
	Chinese	285/1279	1.16 (0.99, 1.35)	0.0660	1.22 (1.04, 1.42)	0.0158*
Normal	IOM	410/2270	Reference		Reference	
	Chinese	587/3049				
High	IOM	148/1192	0.69 (0.56, 0.84)	0.0003	0.67 (0.54, 0.82)	<0.0001*
	Chinese	182/1395	0.68 (0.57, 0.81)	<0.0001	0.65 (0.54, 0.78)	<0.0001*
**Underweight (<18.5 kg/m^2)**						
Low GWG	IOM	-	-	-	-	-
	Chinese	52/118	1.74 (1.17, 2.60)	0.0065	2.10 (1.38, 3.20)	0.0005 §
Normal GWG	IOM	-	-	-	-	-
	Chinese	90/356	Reference			
High GWG	IOM	-	-	-	-	-
	Chinese	30/169	0.70 (0.45, 1.10)	0.1252	0.70 (0.44, 1.11)	0.1244 §
**Normal (18.5~23.9 kg/m^2)**						
Low GWG	IOM	290/1387	1.09 (0.92, 1.29)	0.3351	1.15 (0.97, 1.34)	0.1218 §
	Chinese	201/937	1.11 (0.92, 1.33)	0.2667	1.17 (0.97, 1.40)	0.1087 §
Normal GWG	IOM	365/1897	Reference		Reference	
	Chinese	454/2347				
High GWG	IOM	136/1054	0.67 (0.54, 0.83)	0.0002	0.65 (0.53, 0.80)	<0.0001 §
	Chinese	136/1054	0.68 (0.54, 0.82)	0.0001	0.74 (0.40, 1.36)	<0.0001 §

**Overweight and obese (>=24 kg/m²)**						
Low GWG	IOM	69/94	1.22 (0.76, 1.96)	0.4115	1.29 (0.79, 2.09)	0.3077 §
	Chinese	66/89	1.19 (0.71, 1.87)	0.5754	1.17 (0.71, 1.91)	0.5336 §
Normal GWG	IOM	118/185	Reference		Reference	
	Chinese	116/171				
High GWG	IOM	53/72	0.72 (0.37, 1.40)	0.3353	0.72 (0.37, 1.41)	0.3362 §
	Chinese	58/91	0.75 (0.41, 1.37)	0.3459	0.67 (0.36, 1.24)	0.2011 §

Abbreviations: OR, odds ratio; CI: confidence interval; SGA, small for gestational age; AGA, appropriate for gestational age.

* Adjusted for maternal delivery age (continuous), education level, parity, gravidity, pre-pregnancy BMI, sex of twin infants, and gestational weeks.

§ Adjusted for maternal delivery age (continuous), education level, parity, gravidity, sex of twin infants, and gestational weeks.
